# Supplementary material for: Application of decision analytic modelling to cardiovascular disease prevention in Sub-Saharan Africa: a systematic review
Source: Commun Med (Lond). 2025 Feb 22;5:46. doi: 10.1038/s43856-025-00772-3 (PMC11847006; doi:10.1038/s43856-025-00772-3)
Supplement: Supplementary file 2 — Supplementary Information [file 43856_2025_772_MOESM2_ESM.pdf]

Application of Decision Analytic Modelling to Cardiovascular Disease Prevention in Sub-Saharan Africa: A Systematic Review

James Odhiambo Oguta[1†], Penny Breeze[1], Elvis Wambiya[1], Peter Kibe[2], Catherine Akoth[1], Peter Otieno[2], Peter J. Dodd[1]

1. Sheffield Centre for Health and Related Research, Division of Population Health, School of Medicine and Population Health, Sheffield, United Kingdom.
2. African Population and Health Research Center (APHRC), Nairobi, Kenya.

Supplementary Methods

MEDLINE Search Strategy

Ovid MEDLINE(R) and Epub Ahead of Print, In-Process, In-Data-Review & Other Non-Indexed Citations and Daily <1946 to September 12, 2023>

1 ((decision analytic or decision or cost effectiveness or "extended cost effectiveness" or "distributional cost effectiveness" or "distributional impact" or cost-effective* or microsim* or simulation or cost utility or cost-utility or cost minimi#ation or cost-minimi#ation or Markov or markov cohort or state transition or dynamic or agent-based or mathematical or cost benefit or cost-benefit or system dynamic or systems dynamic or agent based or discrete event simulation or discrete-event* or discrete events or cost of illness or individual patient level simulation or individual patient level) adj model*).tw. 102748

2 (decision adj1 (tree$ or analy$ or model$)).tw. 26836

3 exp "Costs and Cost Analysis"/ 266524

4 exp Cost-Benefit Analysis/ 93173

5 (economic adj (evaluation* or impact)).tw. 27256

6 "financial impact".tw. 2715

7 1 or 2 or 3 or 4 or 5 or 6 389900

8 ("cardiovascular disease" or "heart disease" or stroke or "myocardial infarction" or "myocardial ischaemia" or "transient ischemic attack" or "ischemic attack" or "cerebrovascular disease" or "cerebrovascular accident" or CVA or IHD or CVD or CHD or "cardiovascular event" or angina or "angina pectoris" or "heart attack" or "ischemic heart disease*" or "coronary heart disease" or "coronary disease" or "heart failure" or "acute coronary syndrome" or "peripheral vascular disease" or "Peripheral Vascular diseases" or "atrial fibrillation").tw. 1063014

9 exp Heart Diseases/ 1276479

10 exp Cardiovascular Diseases/ 2735336

11 8 or 9 or 10 3030748

12 (prevent* or control or "prevention and control" or "primary prevention" or "secondary prevention" or "cardiovascular risk" or "risk factor" or lifestyle or behaviour or diet or "diet therapy" or food or "food therapy" or hypertension or "blood pressure" or smoking or tobacco or alcohol or "alcohol consumption" or "physical activity" or exercise or obesity or obese or overweight or "body mass index" or BMI or weight or salt or "salt reduction" or dyslipidaemia or "lipid lowering" or cholesterol or fat or tax or taxation or advertising or counselling or "diet advice" or "health education" or "patient education" or screening or "sugar sweetened beverages" or "less healthy food and drink" or HFSS or "soda tax" or "sugar tax" or "dietary approaches to stop hypertension" or DASH).tw. 8302978

13 exp Primary Prevention/ 183744

14 exp Secondary Prevention/ 22715

15 (food adj (reformulation or junk or unhealthy or discretionary or confectionery or label* or promotion or marketing or policy or sponsorship or licensing)).tw. 2883

16 exp Diet/ 331135

17 exp Diet Therapy/ 62695

18 16 or 17 339803

19 12 or 13 or 14 or 15 or 16 or 17 8512789

20 (angola or benin or botswana or "Burkina Faso" or burundi or "Cabo Verde" or cameroon or "Central African Republic" or chad or comoros or "Democratic Republic of Congo" or congo or "Cote d'Ivoire" or "Ivory Coast" or djibouti or "Equatorial Guinea" or eritrea or ethiopia or gabon or "The Gambia" or ghana or guinea or "Guinea Bissau" or kenya or lesotho or liberia or madagascar or malawi or mali or mauritania or mauritius or mozambique or namibia or niger or

nigeria or rwanda or "Sao Tome and Principe" or senegal or "Sierra Leone" or somalia or "South Africa" or "South Sudan" or sudan or Eswatini or tanzania or togo or uganda or zambia or zimbabwe or africa or "sub-Saharan Africa" or "sub saharan africa" or "low and middle-income countr$" or "low-income countr$" or "Low or middle-income countr$" or "developing country" or "underdeveloped country" or resource-limited).tw. 491367

21 7 and 11 and 19 and 20 473

22 limit 21 to (english language and humans and "remove preprint records") 441

EMBASE Search Strategy

Embase <1974 to 2023 Week 36>

1 ((decision analytic or decision or cost effectiveness or "extended cost effectiveness" or "distributional cost effectiveness" or "distributional impact" or cost-effective* or microsim* or simulation or cost utility or cost-utility or cost minimi#ation or cost-minimi#ation or Markov or markov cohort or state transition or dynamic or agent-based or mathematical or cost benefit or cost-benefit or system dynamic or systems dynamic or agent based or discrete event simulation or discrete-event* or discrete events or cost of illness or individual patient level simulation or individual patient level) adj model*).tw. 127908

2 exp "cost benefit analysis"/ 95038

3 exp "cost utility analysis"/ 12400

4 exp "cost minimization analysis"/ 4015

5 exp "cost effectiveness analysis"/ 182990

6 exp "program cost effectiveness"/ 1063

7 exp "health care cost"/ 343252

8 exp "cost"/ 407477

9 exp "societal cost"/ 444

10 exp "cost of illness"/ 21367

11 (economic adj (evaluation* or impact)).tw. 39160

12 "financial impact".tw. 4645

13 1 or 2 or 3 or 4 or 5 or 6 or 7 or 8 or 9 or 10 or 11 or 12 743539

14 ("cardiovascular disease" or "heart disease" or stroke or "myocardial infarction" or "myocardial ischaemia" or "transient ischemic attack" or "ischemic attack" or "cerebrovascular disease" or "cerebrovascular accident" or CVA or IHD or CVD or CHD or "cardiovascular event" or angina or "angina pectoris" or "heart attack" or "ischemic heart disease*" or "coronary heart disease" or "coronary disease" or "heart failure" or "acute coronary syndrome" or "peripheral vascular disease" or "Peripheral Vascular diseases" or "atrial fibrillation").tw. 1616999

15 exp heart disease/ 2200014

16 exp cardiovascular disease/ 4966577

17 14 or 15 or 16 5175965

18 (prevent* or control or "prevention and control" or "primary prevention" or "secondary prevention" or "cardiovascular risk" or "risk factor" or lifestyle or behaviour or diet or "diet therapy" or food or "food therapy" or hypertension or "blood pressure" or smoking or tobacco or alcohol or "alcohol consumption" or "physical activity" or exercise or obesity or obese or overweight or "body mass index" or BMI or weight or salt or "salt reduction" or dyslipidaemia or "lipid lowering" or cholesterol or fat or tax or taxation or advertising or counselling or "diet advice" or "health education" or "patient education" or screening or "sugar sweetened beverages" or "less healthy food and drink" or HFSS or "soda tax" or "sugar tax" or "dietary approaches to stop hypertension" or DASH).tw. 10844014

19 exp primary prevention/ or exp secondary prevention/ or exp tertiary prevention/ or exp heart infarction prevention/ or exp prevention/ or exp "prevention and control"/ or exp embolism prevention/ or exp prevention study/ 2616310

20 (food adj (reformulation or junk or unhealthy or discretionary or confectionery or label* or promotion or marketing or policy or sponsorship or licensing)).tw. 3861

21 exp diet therapy/ 416580

22 18 or 19 or 20 or 21 12337402

23 (angola or benin or botswana or "Burkina Faso" or burundi or "Cabo Verde" or cameroon or "Central African Republic" or chad or comoros or "Democratic Republic of Congo" or congo or "Cote d'Ivoire" or "Ivory Coast" or djibouti or "Equatorial Guinea" or eritrea or ethiopia or gabon or "The Gambia" or ghana or guinea or "Guinea Bissau" or kenya or lesotho or liberia or madagascar or malawi or mali or mauritania or mauritius or mozambique or namibia or niger or nigeria or rwanda or "Sao Tome and Principe" or senegal or "Sierra Leone" or somalia or "South Africa" or "South Sudan" or sudan or Eswatini or tanzania or togo or uganda or zambia or

zimbabwe or africa or "sub-Saharan Africa" or "sub saharan africa" or "low and middle-income countr$" or "low-income countr$" or "Low or middle-income countr$" or "developing country" or "underdeveloped country" or resource-limited).tw. 550644

24 13 and 17 and 22 and 23 1123

25 limit 24 to (human and english language) 1052

26 limit 25 to (article or article in press or books or chapter) 604

27 limit 26 to "remove preprint records" 604

28 limit 27 to "remove medline records" 113

EconLit Search Strategy

Econlit <1886 to October 12, 2023>

1 ((decision analytic or decision or cost effectiveness or "extended cost effectiveness" or "distributional cost effectiveness" or "distributional impact" or cost-effective* or microsim* or simulation or cost utility or cost-utility or cost minimi#ation or cost-minimi#ation or Markov or markov cohort or state transition or dynamic or agent-based or mathematical or cost benefit or cost-benefit or system dynamic or systems dynamic or agent based or discrete event simulation or discrete-event* or discrete events or cost of illness or individual patient level simulation or individual patient level) adj model*).tw. 14774

2 (decision adj1 (tree$ or analy$ or model$)).tw. 2387

3 (economic adj (evaluation* or impact)).tw. 5586

4 "financial impact".tw. 303

5 ("cardiovascular disease" or "heart disease" or stroke or "myocardial infarction" or "myocardial ischaemia" or "transient ischemic attack" or "ischemic attack" or "cerebrovascular disease" or "cerebrovascular accident" or CVA or IHD or CVD or CHD or "cardiovascular event" or angina or "angina pectoris" or "heart attack" or "ischemic heart disease*" or "coronary heart disease" or "coronary disease" or "heart failure" or "acute coronary syndrome" or "peripheral vascular disease" or "Peripheral Vascular diseases" or "atrial fibrillation").tw. 1330

6 (prevent* or control or "prevention and control" or "primary prevention" or "secondary prevention" or "cardiovascular risk" or "risk factor" or lifestyle or behaviour or diet or "diet therapy" or food or "food therapy" or hypertension or "blood pressure" or smoking or tobacco or alcohol or "alcohol consumption" or "physical activity" or exercise or obesity or obese or overweight or "body mass index" or BMI or weight or salt or "salt reduction" or dyslipidaemia or "lipid lowering" or cholesterol or fat or tax or taxation or advertising or counselling or "diet advice" or "health education" or "patient education" or screening or "sugar sweetened beverages" or "less healthy food and drink" or HFSS or "soda tax" or "sugar tax" or "dietary approaches to stop hypertension" or DASH).tw. 233092

7 (food adj (reformulation or junk or unhealthy or discretionary or confectionery or label* or promotion or marketing or policy or sponsorship or licensing)).tw. 998

8 1 or 2 or 3 or 4 21891

9 6 or 7 233092

10 5 and 8 and 9 51

11 limit 10 to (journal articles and english and journal articles) 46

PsycInfo Search Strategy

APA PsycInfo <1806 to September Week 1 2023>

1 ((decision analytic or decision or cost effectiveness or "extended cost effectiveness" or "distributional cost effectiveness" or "distributional impact" or cost-effective* or microsim* or simulation or cost utility or cost-utility or cost minimi#ation or cost-minimi#ation or Markov or markov cohort or state transition or dynamic or agent-based or mathematical or cost benefit or cost-benefit or system dynamic or systems dynamic or agent based or discrete event simulation or discrete-event* or discrete events or cost of illness or individual patient level simulation or individual patient level) adj model*).tw. 14808

2 (decision adj1 (tree$ or analy$ or model$)).tw. 5415

3 exp "Costs and Cost Analysis"/ 49753

4 exp Health Care Economics/ 1218

5 exp Economics/ 92950

6 exp Health Care Costs/ 25942

7 1 or 2 or 3 or 4 or 5 or 6 137323

8 ("cardiovascular disease" or "heart disease" or stroke or "myocardial infarction" or "myocardial ischaemia" or "transient ischemic attack" or "ischemic attack" or "cerebrovascular disease" or "cerebrovascular accident" or CVA or IHD or CVD or CHD or "cardiovascular event" or angina or "angina pectoris" or "heart attack" or "ischemic heart disease*" or "coronary heart disease" or "coronary disease" or "heart failure" or "acute coronary syndrome" or "peripheral vascular disease" or "Peripheral Vascular diseases" or "atrial fibrillation").tw. 70022

9 (prevent* or control or "prevention and control" or "primary prevention" or "secondary prevention" or "cardiovascular risk" or "risk factor" or lifestyle or behaviour or diet or "diet therapy" or food or "food therapy" or hypertension or "blood pressure" or smoking or tobacco or alcohol or "alcohol consumption" or "physical activity" or exercise or obesity or obese or overweight or "body mass index" or BMI or weight or salt or "salt reduction" or dyslipidaemia or "lipid lowering" or cholesterol or fat or tax or taxation or advertising or counselling or "diet advice" or "health education" or "patient education" or screening or "sugar sweetened beverages" or "less healthy food and drink" or HFSS or "soda tax" or "sugar tax" or "dietary approaches to stop hypertension" or DASH).tw. 1295498

10 exp Prevention/ 75576

11 (food adj (reformulation or junk or unhealthy or discretionary or confectionery or label* or promotion or marketing or policy or sponsorship or licensing)).tw. 955

12 exp Diets/ 20483

13 exp Cardiovascular Disorders/ or exp Cerebrovascular Accidents/ or exp Heart Disorders/ 72207

14 9 or 10 or 11 or 12 1304078

15 8 or 13 96736

16 (angola or benin or botswana or "Burkina Faso" or burundi or "Cabo Verde" or cameroon or "Central African Republic" or chad or comoros or "Democratic Republic of Congo" or congo or "Cote d'Ivoire" or "Ivory Coast" or djibouti or "Equatorial Guinea" or eritrea or ethiopia or gabon or "The Gambia" or ghana or guinea or "Guinea Bissau" or kenya or lesotho or liberia or madagascar or malawi or mali or mauritania or mauritius or mozambique or namibia or niger or nigeria or rwanda or "Sao Tome and Principe" or senegal or "Sierra Leone" or somalia or "South Africa" or "South Sudan" or sudan or Eswatini or tanzania or togo or uganda or zambia or zimbabwe or africa or "sub-Saharan Africa" or "sub saharan africa" or "low and middle-income countr$" or "low-income countr$" or "Low or middle-income countr$" or "developing country" or "underdeveloped country" or resource-limited).tw. 67073

17 7 and 14 and 15 and 16 20

18 limit 17 to (human and english language) 18

Scopus Search Strategy

((TITLE-ABS-KEY("decision analytic" or "decision model" or "cost effectiveness" or cost-effective* or "extended cost effectiveness" or "distributional cost effectiveness" or "distributional impact" or microsim* or simulation or "cost utility" or cost-utility or "cost minimi$ation" or cost-minimi$ation or Markov or "Markov cohort" or "state transition" or "dynamic model" or agent-based or "mathematical model" or "cost benefit" or cost-benefit or "system dynamic" or "systems dynamic" or "agent based" or "discrete event simulation" or discrete-event* or "discrete events" or "cost of illness" or "individual patient level simulation" or "individual patient level")) OR (TITLE-ABS-KEY("cost benefit analysis" OR "cost analysis")) OR (TITLE-ABS-KEY(economic W/2 (evaluation* OR impact))) OR (TITLE-ABS-KEY("financial impact")) OR (TITLE-ABS-KEY(decision W/2 (tree* OR analy* OR model*)))) AND (TITLE-ABS-KEY("cardiovascular disease" OR "heart disease" OR stroke OR "myocardial infarction" OR "myocardial ischaemia" OR "transient ischemic attack" OR "ischemic attack" OR "cerebrovascular disease" OR "cerebrovascular accident" OR cva OR ihd OR cvd OR chd OR "cardiovascular event" OR angina OR "angina pectoris" OR "heart attack" OR "ischemic heart disease*" OR "coronary heart disease" OR "coronary disease" OR "heart failure" OR "acute coronary syndrome" OR "peripheral vascular disease" OR "Peripheral Vascular diseases" OR "atrial fibrillation")) AND ((TITLE-ABS-KEY(prevent* or control or "prevention and control" or "primary prevention" or "secondary prevention" or "cardiovascular risk" or "risk factor" or lifestyle or behaviour or diet or "diet therapy" or food or "food therapy" or hypertension or "blood pressure" or smoking or tobacco or alcohol or "alcohol consumption" or "physical activity" or exercise or obesity or obese or overweight or "body mass index" or BMI or weight or salt or "salt reduction" or dyslipidaemia or "lipid lowering" or cholesterol or fat or tax or taxation or advertising or counselling or "diet advice" or "health education" or "patient education" or screening or "sugar sweetened beverages" or "less healthy food and drink" or HFSS or "soda tax" or "sugar tax" or "dietary approaches to stop hypertension" or DASH)) OR (TITLE-ABS-KEY(food w/2 (reformulation or junk or unhealthy or discretionary or confectionery or label* or promotion or marketing or policy or sponsorship or licensing)))) AND (TITLE-ABS-KEY(angola or benin or botswana or "Burkina Faso" or burundi or "Cabo Verde" or cameroon or "Central African Republic" or chad or comoros or "Democratic Republic of Congo" or congo or "Cote d'Ivoire" or "Ivory Coast" or djibouti or "Equatorial Guinea" or eritrea or ethiopia or gabon or "The Gambia" or ghana or guinea or "Guinea Bissau" or kenya or lesotho or liberia or madagascar or malawi or mali or mauritania or mauritius or mozambique or namibia or niger or nigeria or rwanda or "Sao Tome and Principe" or senegal or "Sierra Leone" or

somalia or "South Africa" or "South Sudan" or sudan or Eswatini or tanzania or togo or uganda or zambia or zimbabwe or africa or "sub-Saharan Africa" or "sub saharan africa" or "low and middle-income countr$" or "low-income countr$" or "Low or middle-income countr$" or "developing country" or "underdeveloped country" or resource-limited)) AND (EXCLUDE (DOCTYPE,"cp") OR EXCLUDE (DOCTYPE,"ed") OR EXCLUDE (DOCTYPE,"no") OR EXCLUDE (DOCTYPE,"le") OR EXCLUDE (DOCTYPE,"cr") OR EXCLUDE (DOCTYPE,"sh") OR EXCLUDE (DOCTYPE,"er")) AND (LIMIT-TO (LANGUAGE,"English")) AND (LIMIT-TO (EXACTKEYWORD,"Human") OR LIMIT-TO (EXACTKEYWORD,"Humans"))

402

Web of Science Search Strategy

Web of Science Search Strategy (v0.1)
Database: All Databases
Entitlements:
- WOS: 1900 to 2023
- BCI: 1926 to 2023
- BIOSIS: 1969 to 2023
- CCC: 1998 to 2023
- DRCI: 1900 to 2023
- DIIDW: 1966 to 2023
- KJD: 1980 to 2023
- MEDLINE: 1950 to 2023
- PPRN: 1991 to 2023
- PQDT: 1637 to 2023
- SCIELO: 2002 to 2023
- ZOOREC: 1864 to 2023

Searches:

1: TS=("decision analytic" or "decision model" or "cost effectiveness" or cost-effective* or "extended cost effectiveness" or "distributional cost effectiveness" or "distributional impact" or microsim* or simulation or "cost utility" or cost-utility or "cost minimi$ation" or cost-minimi$ation or Markov or "Markov cohort" or "state transition" or "dynamic model" or agent-based or "mathematical model" or "cost benefit" or cost-benefit or "system dynamic" or "systems dynamic" or "agent based" or "discrete event simulation" or discrete-event* or "discrete events" or "cost of illness" or "individual patient level simulation" or "individual patient level") and Preprint Citation Index (Exclude – Database) Results: 6714698

2: TS=(decision near/5 (tree* or analy* or model*)) and Preprint Citation Index (Exclude – Database) Results: 314034

3: TS=("cost benefit analysis" or "cost analysis") and Preprint Citation Index (Exclude – Database) Results: 205944

4: TS=(economic near/5 (evaluation* or impact)) and Preprint Citation Index (Exclude – Database) Results: 173078

5: TS=("financial impact") and Preprint Citation Index (Exclude – Database) Results: 6004

6: #5 OR #4 OR #3 OR #2 OR #1 and Preprint Citation Index (Exclude – Database) Results: 7151881

7: TS=("cardiovascular disease" or "heart disease" or stroke or "myocardial infarction" or "myocardial ischaemia" or "transient ischemic attack" or "ischemic attack" or "cerebrovascular disease" or "cerebrovascular accident" or CVA or IHD or CVD or CHD or "cardiovascular event" or angina or "angina pectoris" or "heart attack" or "ischemic heart disease*" or "coronary heart disease" or "coronary disease" or "heart failure" or "acute coronary syndrome" or "peripheral vascular disease" or "Peripheral Vascular diseases" or "atrial fibrillation") and Preprint Citation Index (Exclude – Database) Results: 3093758

8: TS=(prevent* or control or "prevention and control" or "primary prevention" or "secondary prevention" or "cardiovascular risk" or "risk factor" or lifestyle or behaviour or diet or "diet therapy" or food or "food therapy" or hypertension or "blood pressure" or smoking or tobacco or alcohol or "alcohol consumption" or "physical activity" or exercise or obesity or obese or overweight or "body mass index" or BMI or weight or salt or "salt reduction" or dyslipidaemia or "lipid lowering" or cholesterol or fat or tax or taxation or advertising or counselling or "diet advice"

or "health education" or "patient education" or screening or "sugar sweetened beverages" or "less healthy food and drink" or HFSS or "soda tax" or "sugar tax" or "dietary approaches to stop hypertension" or DASH) and Preprint Citation Index (Exclude – Database) Results: 57236098

9: TS=(food near/5 (reformulation or junk or unhealthy or discretionary or confectionery or label* or promotion or marketing or policy or sponsorship or licensing)) and Preprint Citation Index (Exclude – Database) Results: 82987

10: #9 OR #8 and Preprint Citation Index (Exclude – Database) Results: 57236098

11: 1: TS=(angola or benin or botswana or "Burkina Faso" or burundi or "Cabo Verde" or cameroon or "Central African Republic" or chad or comoros or "Democratic Republic of Congo" or congo or "Cote d'Ivoire" or "Ivory Coast" or djibouti or "Equatorial Guinea" or eritrea or ethiopia or gabon or "The Gambia" or ghana or guinea or "Guinea Bissau" or kenya or lesotho or liberia or madagascar or malawi or mali or mauritania or mauritius or mozambique or namibia or niger or nigeria or rwanda or "Sao Tome and Principe" or senegal or "Sierra Leone" or somalia or "South Africa" or "South Sudan" or sudan or Eswatini or tanzania or togo or uganda or zambia or zimbabwe or africa or "sub-Saharan Africa" or "sub saharan africa" or "low and middle-income countr$" or "low-income countr$" or "Low or middle-income countr$" or "developing country" or "underdeveloped country" or resource-limited) and Preprint Citation Index (Exclude – Database) Results: 2129215

12: #11 AND #10 AND #7 AND #6 and Preprint Citation Index (Exclude – Database) Results: 1001

13: #11 AND #10 AND #7 AND #6 and Preprint Citation Index (Exclude – Database) and Retracted Publication or News or Patent or Case Report or Early Access or Data Study or Letter or Book or Editorial Material or Data Set or Dissertation Thesis or Abstract or Meeting or Clinical Trial (Exclude – Document Types) Results: 811

14: #11 AND #10 AND #7 AND #6 and Preprint Citation Index (Exclude – Database) and Retracted Publication or News or Patent or Case Report or Early Access or Data Study or Letter or Book or Editorial Material or Data Set or Dissertation Thesis or Abstract or Meeting or Clinical Trial (Exclude – Document Types) and English (Languages) Results: 756

CINAHL Search Strategy

S20 S7 AND S11 AND S17 AND S18 Expanders - Apply equivalent subjects
Search modes - Boolean/Phrase Interface - EBSCOhost Research Databases
Search Screen - Advanced Search
Database - CINAHL 257

S19 S7 AND S11 AND S17 AND S18 Expanders - Apply equivalent subjects
Search modes - Boolean/Phrase Interface - EBSCOhost Research Databases
Search Screen - Advanced Search
Database - CINAHL 257

S18 angola or benin or botswana or "Burkina Faso" or burundi or "Cabo Verde" or cameroon or "Central African Republic" or chad or comoros or "Democratic Republic of Congo" or congo or "Cote d'Ivoire" or "Ivory Coast" or djibouti or "Equatorial Guinea" or eritrea or ethiopia or gabon or "The Gambia" or ghana or guinea or "Guinea Bissau" or kenya or lesotho or liberia or madagascar or malawi or mali or mauritania or mauritius or mozambique or namibia or niger or nigeria or rwanda or "Sao Tome and Principe" or senegal or "Sierra Leone" or somalia or "South Africa" or "South Sudan" or sudan or Eswatini or tanzania or togo or uganda or zambia or zimbabwe or africa or "sub-Saharan Africa" or "sub saharan africa" or "low and middle-income countr*" or "low-income countr*" or "Low or middle-income countr*" or "developing country" or "underdeveloped country" or resource-limited Expanders - Apply equivalent subjects

Search modes - Boolean/Phrase Interface - EBSCOhost Research Databases
Search Screen - Advanced Search
Database - CINAHL 145,298

S17 S12 OR S13 OR S14 OR S15 OR S16 Expanders - Apply equivalent subjects
Search modes - Boolean/Phrase Interface - EBSCOhost Research Databases
Search Screen - Advanced Search
Database - CINAHL 2,836,730

S16 (MH "Diet+") OR (MH "Diet, Fat-Restricted") OR (MH "Diet, Low Carbohydrate") OR (MH "Diet, Sodium-Restricted") OR (MH "Diet, Gluten-Free") OR (MH "Restricted Diet") OR (MH "Diet, Reducing") Expanders - Apply equivalent subjects
Search modes - Boolean/Phrase Interface - EBSCOhost Research Databases
Search Screen - Advanced Search
Database - CINAHL 142,116

S15 food n5 (reformulation or junk or unhealthy or discretionary or confectionery or label* or promotion or marketing or policy or sponsorship or licensing) Expanders - Apply equivalent subjects
Search modes - Boolean/Phrase Interface - EBSCOhost Research Databases
Search Screen - Advanced Search
Database - CINAHL 12,013

S14 diet n5 (mediterranean or "low carbohydrate" or fad or atkins or intermittent or vegetarian or vegan) Expanders - Apply equivalent subjects
Search modes - Boolean/Phrase Interface - EBSCOhost Research Databases
Search Screen - Advanced Search
Database - CINAHL 10,375

S13 (MH "Cardiovascular Risk Factors+") Expanders - Apply equivalent subjects
Search modes - Boolean/Phrase Interface - EBSCOhost Research Databases
Search Screen - Advanced Search
Database - CINAHL 29,814

S12 prevent* or control or "prevention and control" or "primary prevention" or "secondary prevention" or "cardiovascular risk" or "risk factor" or lifestyle or behaviour or diet or "diet therapy" or food or "food therapy" or hypertension or "blood pressure" or smoking or tobacco or alcohol or "alcohol consumption" or "physical activity" or exercise or obesity or obese or overweight or "body mass index" or BMI or weight or salt or "salt reduction" or dyslipidaemia or "lipid lowering" or cholesterol or fat or tax or taxation or advertising or counselling or "diet advice" or "health education" or "patient education" or screening or "sugar sweetened beverages" or "less healthy food and drink" or HFSS or "soda tax" or "sugar tax" or "dietary approaches to stop hypertension" or DASH Expanders - Apply equivalent subjects
Search modes - Boolean/Phrase Interface - EBSCOhost Research Databases
Search Screen - Advanced Search
Database - CINAHL 2,830,290

S11 S8 OR S9 OR S10 Expanders - Apply equivalent subjects
Search modes - Boolean/Phrase Interface - EBSCOhost Research Databases
Search Screen - Advanced Search
Database - CINAHL 575,529

S10 (MH "Cardiovascular Diseases") OR (MH "Peripheral Vascular Diseases") Expanders - Apply equivalent subjects
Search modes - Boolean/Phrase Interface - EBSCOhost Research Databases
Search Screen - Advanced Search
Database - CINAHL 66,483

S9 (MH "Myocardial Ischemia+") OR (MH "Pulmonary Heart Disease") OR (MH "Heart Valve Diseases+") OR (MH "Coronary Disease+") OR (MH "Heart Diseases+") OR (MH "Rheumatic Heart Disease") Expanders - Apply equivalent subjects
Search modes - Boolean/Phrase Interface - EBSCOhost Research Databases
Search Screen - Advanced Search
Database - CINAHL 313,565

S8 "cardiovascular disease" or "heart disease" or stroke or "myocardial infarction" or "myocardial ischaemia" or "transient ischemic attack" or "ischemic attack" or "cerebrovascular disease" or "cerebrovascular accident" or CVA or IHD or CVD or CHD or "cardiovascular event" or angina or "angina pectoris" or "heart attack" or "ischemic heart disease*" or "coronary heart disease" or "coronary disease" or "heart failure" or "acute coronary syndrome" or "peripheral vascular disease" or "Peripheral Vascular diseases" or "atrial fibrillation" Expanders - Apply equivalent subjects
Search modes - Boolean/Phrase Interface - EBSCOhost Research Databases
Search Screen - Advanced Search
Database - CINAHL 453,732

S7 S1 OR S2 OR S3 OR S4 OR S5 OR S6 Expanders - Apply equivalent subjects
Search modes - Boolean/Phrase Interface - EBSCOhost Research Databases
Search Screen - Advanced Search
Database - CINAHL 488,064

S6 "financial impact" Expanders - Apply equivalent subjects
Search modes - Boolean/Phrase Interface - EBSCOhost Research Databases
Search Screen - Advanced Search
Database - CINAHL 1,331

S5 economic n5 (evaluation* or impact) Expanders - Apply equivalent subjects
Search modes - Boolean/Phrase Interface - EBSCOhost Research Databases
Search Screen - Advanced Search
Database - CINAHL 13,678

S4 (MH "Economics, Pharmaceutical") Expanders - Apply equivalent subjects
Search modes - Boolean/Phrase Interface - EBSCOhost Research Databases
Search Screen - Advanced Search
Database - CINAHL 2,392

S3 decision n5 (tree* or analy* or model*) Expanders - Apply equivalent subjects
Search modes - Boolean/Phrase Interface - EBSCOhost Research Databases
Search Screen - Advanced Search
Database - CINAHL 18,329

S2 (MH "Costs and Cost Analysis+") OR (MH "Health Care Costs+") OR (MH "Health Facility Costs") OR (MH "Cost Benefit Analysis") OR (MH "Cost Savings") OR (MH "Economic Aspects of Illness") OR (MH "Nursing Costs") Expanders - Apply equivalent subjects
Search modes - Boolean/Phrase Interface - EBSCOhost Research Databases
Search Screen - Advanced Search
Database - CINAHL 141,590

S1 “decision analytic” or decision or “cost effectiveness” or "extended cost effectiveness" or "distributional cost effectiveness" or "distributional impact" or cost-effective* or microsim* or simulation or "cost utility" or cost-utility or "cost minimisation" or cost-minimisation or Markov or "Markov cohort" or "state transition" or "dynamic model" or agent-based or "mathematical model" or "cost benefit" or cost-benefit or "system dynamic" or "systems dynamic" or "agent based" or "discrete event simulation" or discrete-event* or "discrete events" or "cost of illness" or "individual patient level simulation" or "individual patient level" Expanders - Apply equivalent subjects
Search modes - Boolean/Phrase Interface - EBSCOhost Research Databases

Search Screen - Advanced Search
Database - CINAHL 397,082
