# Supplementary material for: Application of decision analytic modelling to cardiovascular disease prevention in Sub-Saharan Africa: a systematic review
Source: Commun Med (Lond). 2025 Feb 22;5:46. doi: 10.1038/s43856-025-00772-3 (PMC11847006; doi:10.1038/s43856-025-00772-3)
Supplement: Supplementary file 3 — Description of Additional Supplementary Files [file 43856_2025_772_MOESM3_ESM.pdf]

Description of Additional Supplementary Files

File name: Supplementary Data 1

File description: Tables 2-4 and Supplementary Table 1
